# Influence of facial shape on perceived attractiveness

**DOI:** 10.1186/s40510-026-00617-2

**Published:** 2026-03-25

**Authors:** Nikolaos Gkantidis, Sven Stucki, Mohammed Ghamri, Demetrios Halazonetis, Georgios Kanavakis

**Affiliations:** 1 Department of Orthodontics and Dentofacial Orthopedics, School of Dental Medicine, University of Bern, Bern, Switzerland; 2 Jeddah Second Health Cluster, Jeddah, Saudi Arabia; 3 Department of Orthodontics, School of Dentistry, National and Kapodistrian University of Athens, Athens, Greece; 4 Department of Orthodontics and Dentofacial Orthopedics, Tufts University School of Dental Medicine, Tufts University, Medford, USA

**Keywords:** Esthetics, Face/anatomy and histology, Humans, Observers, Physical attractiveness, Three-dimensional imaging

## Abstract

**Background/objectives:**

Facial attractiveness is often regarded as subjective, yet perceptions are strongly influenced by cultural, social, and biological factors. This study aimed to investigate whether and to what extent three-dimensional facial shape is associated with attractiveness ratings assigned by external evaluators, and whether these associations differ between males and females.

**Methods:**

A total of 601 young adults (393 females; 208 males), aged 21–35 years, were assessed using three-dimensional facial photography. Facial shape was quantified using 29 anatomical curves and 1,021 landmarks analyzed through geometric morphometric methods. Six external examiners (three males, three females), matched in age to the study population, independently rated attractiveness using Visual Analogue Scales (VAS). Multivariate regression models were applied to examine associations between facial shape and attractiveness scores. Analyses were conducted separately for males and females to account for biological differences in facial morphology.

**Results:**

Facial shape was significantly associated with attractiveness ratings in females (η² = 0.075; *P* < 0.001). Higher attractiveness scores were linked to well-balanced vertical facial proportions and a more angular facial appearance, with sharper contours, reduced facial fullness, and mild profile convexity. A fuller and more projected upper lip was also characteristic of higher-rated faces. In males, effect size was comparable (η² = 0.070), but the association did not reach statistical significance (*P* = 0.106). Attractive male faces tended to exhibit an angular and well-defined facial structure, decreased lateral fullness, slightly increased lower facial height, central facial projection, and a straight profile.

**Conclusions:**

Facial shape is associated with externally perceived facial attractiveness, with statistically significant effects observed in females. The findings align with previous research on self-perceived attractiveness and underscore the influence of facial morphology on societal perceptions of attractiveness.

**Supplementary Information:**

The online version contains supplementary material available at 10.1186/s40510-026-00617-2.

## Introduction

The human face is the most important structure that people use to evaluate physical attractiveness [1]. Facial features are key in shaping the initial impression one forms when meeting a person for the first time [2]. First impressions are made in one tenth of a second and have a strong and lasting impact on human relationships [3, 4]. In addition to basic information, such as sex or age, we consciously or unconsciously form opinions about others upon our first visual contact with them. These opinions are related to traits such as trustworthiness, competence, and intelligence [3].

A person that is judged as attractive is often treated more favorably, which may subsequently lead to higher professional success, a more fulfilling social life and more meaningful romantic relationships [5]. Thus, facial attractiveness can greatly impact a person’s quality of life. In addition to its social impact, the appearance of the face is the focus of many professional disciplines involved in the treatment of the craniofacial region [6]. Therefore, the association between facial morphology and facial attractiveness is of broad scientific interest across various fields.

It has been repeatedly demonstrated that there are objective factors influencing the attractiveness of the face, such as symmetry, averageness, and sexual dimorphism [7–10]. Whether a fully symmetric face is perceived as attractive is discussed controversially in the literature, nevertheless it is well accepted that major asymmetries are viewed unfavorably [7, 11]. In regard to sexual facial characteristics, in females a feminine face is considered more attractive, whereas in males the situation is less clear. Some studies support that a slightly feminine face is also perceived as more attractive in males [12–17], while others show that male facial attractiveness is related to masculine facial features [18–21].

In previous investigations, this group has revealed specific associations between facial morphology and the self-perception of facial attractiveness [22]. In females, a face of decreased width, with a fuller anterior part at its lower third, and a slightly pronounced middle forehead and base of the nose, was perceived as more attractive. In males, a prominent chin, flat cheeks and zygomas, as well as pronounced eyebrow ridges, nose, and middle forehead were perceived as more attractive. Furthermore, smile dimensions have been linked to self-perceived smile attractiveness, reinforcing the notion that facial morphology plays a critical role in shaping both facial and smile attractiveness [23].

Although self-perceived facial attractiveness has recently gained interest [22–25], most studies have examined attractiveness as judged by external raters [26–31], with results often being heterogeneous and sometimes contradictory. Further insight could be gained by assessing the association between facial morphology and attractiveness as perceived by others, particularly through three-dimensional facial analysis and geometric morphometric methods, which enhance objectivity and reliability. Also, certain evidence suggests overlap between features linked to self-perception and external ratings [24], but the extent to which these characteristics align remains unclear. In this study, we first investigated the association between facial shape and externally assessed attractiveness in a large sample of young adults, and secondarily compared external ratings with self-perceived ratings using previously published data [22]. Given the well-established sexual dimorphism in human facial morphology [22], including differences in facial width, projection patterns, soft tissue distribution, and hormonal influences on craniofacial development [7, 8, 32], combining males and females into a single model may obscure biologically meaningful variation. Moreover, attractiveness standards are partly sex-specific and may reflect different biological and sociocultural expectations for male and female faces [5, 14]. For these reasons, all analyses were conducted separately for males and females. The findings are expected to improve our understanding of how facial shape influences attractiveness and to highlight potential differences between self- and externally perceived attractiveness.

## Materials and methods

The present study is a cross-sectional, observational investigation, part of a larger project, certain aspects of which have already been published [22, 23, 33]. The study sample and methodology for facial shape assessment have been described elsewhere [22], but the most important information is repeated here in order to facilitate better comprehension and easier reading of the manuscript.

### Ethical approval

The protocol of this investigation was reviewed and approved by the Health Sciences Institutional Review Board (IRB) of Tufts University in Boston, Massachusetts (IRB#: 11181) . All experiments were performed in accordance with relevant guidelines and regulations. Written informed consent was obtained from all participants prior to their inclusion in the study.

### Population

The initial study population consisted of 613 (214 males; 399 females) volunteers, all pre-doctoral medical or dental students at Tufts University in Boston, Massachusetts, who responded to an on-campus advertisement. All participants were young adults (21 to 35 years old) raised in the United States and spoke English as their native language. Sex, ethnicity, and sexuality were self-reported by the participants. Individuals who presented craniofacial syndromes, visible facial deformities or had a history of facial plastic surgery were excluded. After recruitment and participation, data were also excluded on the basis of sexual orientation, due to reported differences in appreciation of facial attractiveness [34]. The final study sample consisted of 601 heterosexual young adults (208 males; 393 females) with various ethnic backgrounds. Demographically, the sample included the following subgroups: 368 White participants (149 males and 219 females), 22 Black participants (3 males and 19 females), 123 Asian participants (39 males and 84 females), 71 South Asian (Indian) participants (14 males and 57 females), and 21 multiracial participants (4 males and 17 females).

### 3D facial image acquisition

A stereophotogrammetry system (3dMD, Atlanta, USA) was used to capture a three-dimensional facial image of each participant in a resting position [35–37]. Subjects were placed at a fixed distance of approximately 100 cm from the camera unit, with their head slightly raised (10 degrees) according to the camera guidelines (upright head position). To ensure standardization, participants were seated on a chair, with their upper body in a comfortable upright position and their back resting on the back of the chair. They were asked to maintain the upright head position, which was also adjusted by an experienced research team member, if needed. In order to acquire the image at rest, subjects were instructed to keep their teeth in slight contact, their lips in a resting position, without straining, and their eyes open without straining the forehead.

### Facial shape definition

**Fig. 1:**
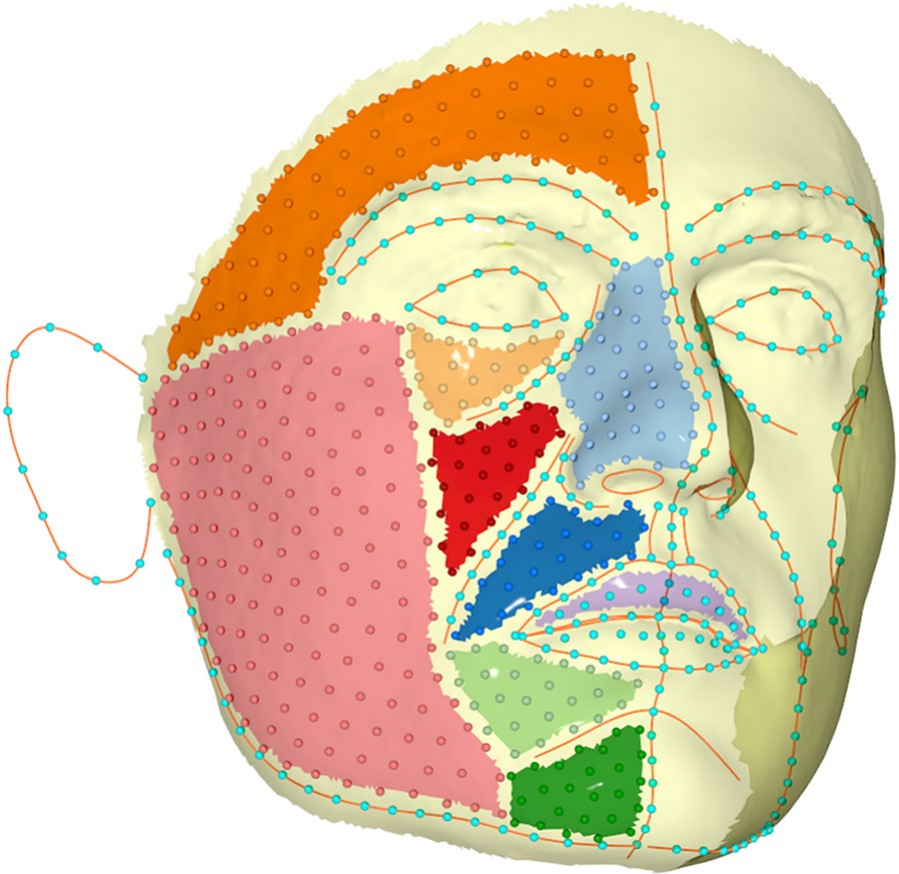
Distribution of all 1,021 landmarks and semi-landmarks on the facial surface. Semi-landmarks were initially positioned to depict various areas of the face but were allowed to slide along curves (curve semi-landmarks) or the surface (surface semi-landmarks) during TPS (Thin Plate Spline) transformation. To avoid clutter, surface semi-landmarks are only depicted on one side of the face. Reprint from Kanavakis et al. [22]

The digitization of the 3D facial surface images and the geometric morphometric methods described below have been reported in a previous publication assessing the association between facial shape and self-perceived facial attractiveness [22]. Facial images were imported in “ViewBox 4.1” software (dHAL Software, Kifissia, Greece), where 1,021 predefined landmarks and semi-landmarks were placed on a reference (template) facial image [38] (Fig. 1). The actual sample images were digitized semi-automatically, first by manually drawing curves on anatomical structures of the face and, subsequently, by automatically adding semi-landmarks at equidistant positions on the curves and at uniform locations on the surface of the face, delimited according to the manually drawn curves and specific fixed landmarks. The latter correspond to points identified by local anatomy, such as the anatomical limits of the eyes (inner and outer canthi) and the mouth (inner and outer stomia); they were manually placed and considered as traditional landmarks that remained fixed throughout all the stages of the analysis (see below). The other curve landmarks, as well as the surface landmarks, were allowed to slide on their corresponding curves or the facial surface, respectively, and were thus treated as semi-landmarks [39]. Fixed landmarks and curve semi-landmarks were placed first and were used to guide the placement of all the surface semi-landmarks according to the reference shape.

After the digitization of all images, sliding of semi-landmarks along their corresponding curve or the entire surface was performed iteratively to minimize TPS (Thin Plate Spline) bending energy between each landmark configuration and the average facial shape of the sample [40]. Three cycles of sliding and re-projecting were repeated until no meaningful change in bending energy was observed. The resulting landmark configurations were considered to be homologous representations of the facial surfaces and comprised the final sample for all following shape analyses. A Generalized Procrustes superimposition was used to superimpose landmark configurations and transform landmark coordinates to Procrustes coordinates [32, 41]. A Principal Component Analysis (PCA) was performed to reduce the number of shape variables (Procrustes coordinates) for subsequent statistical analyses and explore shape variation with shape-space principal components (PCs).

The error of the facial shape definition process has been previously reported and judged as acceptable [22]. The random error was 6.9% and there was no indication of systematic error.

### Assessment of facial shape

The shape variation of the sample has been reported in a previous publication, separately for males and females, due to the significant sexual dimorphism [22]. In the present study, only PCs relevant to the analysis were visualized, by morphing facial shapes using TPS transformations [21]. These were created by warping the surface of one male and one female individual that were both relatively close to the sample average according to each corresponding landmark configuration. In addition, to assess the association between facial shape and facial attractiveness ratings by external evaluators, extreme values of self-perceived facial attractiveness (± 3SD) were also visualized through facial shape morphing. Following each analysis, the morphed 3D surface meshes of the two extreme facial shapes were best-fit approximated through the repeated application of a variant of the iterative closest point (ICP) algorithm [42], under the following software settings: 100% estimated overlap of meshes, matching point to normal plane, exact nearest neighbouring point search, 100% point sampling, 50 iterations, exclude overhangs. Color-coded distance maps were generated to facilitate assessments.

Descriptive terms such as “angularity” and “facial fullness” refer to specific three-dimensional shape characteristics quantified through geometric morphometric analysis. “Angularity” denotes decreased soft tissue prominence, such as in the buccal and zygomatic regions, resulting in more sharply defined underlying skeletal contours, such as those along the mandibular border and zygomatic arches. In contrast, “facial fullness” describes increased soft tissue volume in the mandibular, buccal, and infraorbital regions, leading to smoother contour transitions and reduced skeletal definition.

### Assessment of facial attractiveness

For the purpose of the present investigation, the questionnaire previously completed by the study participants themselves [22] was also completed by 6 external evaluators independently (3 males and 3 females). The external evaluators had similar characteristics to the assessed participants (young adults with health sciences background), did not participate in any other part of the study, and were not informed about the specific study outcome.

Afterwards, the external evaluators were familiarized with viewing 3D images on Viewbox 4 software. Each external evaluator performed a visual assessment of each 3D facial image of all 601 participants, who had previously performed a self-evaluation of their facial attractiveness. Prior to questionnaire fulfilment, each 3D image was viewed from different angles (operator-free decision) and not for longer than a minute. While viewing each 3D facial image, the evaluators completed the study questionnaire on their screen and recorded their answers digitally, in an Excel sheet (Microsoft Excel, Microsoft, Redmond WA, USA). The questionnaires were completed privately, under regular office conditions, and consisted of 5 units, 4 of which assessed psychometrics and personality traits that will be discussed elsewhere. In the present study, data from unit A, which evaluated facial attractiveness, were analysed (Supplementary Fig. 1). Facial attractiveness was assessed using a 100-mm Visual Analogue Scale (VAS), a widely used continuous rating method with demonstrated reliability and robust psychometric properties in facial attractiveness research [43, 44]. To complete the entire questionnaire, a timeframe of about 15 min per image was expected. All study procedures related to the external ratings were performed based on the standardized instructions of an experienced research team member (G.K.) . The validity of the questionnaire used for self-assessments was thoroughly evaluated and confirmed in a previous publication [22]. In the present study, the same questionnaire format was used, with the only modification being that it referred to the assessment of faces viewed on a screen.

### Statistical analyses

Facial attractiveness was described by the average value of the six external attractiveness ratings. Interrater reliability among the six examiners was tested using an average-measures intraclass correlation coefficient, based on a two-way random-effects model with absolute agreement. Comparisons of facial attractiveness scores within the entire sample and within ethnic subgroups were performed with independent t-tests. Due to inherent sexual differences, including sexual dimorphism in facial shape [22], all analyses were performed separately in males and females.

The relationship between facial attractiveness and facial shape was examined using multivariate linear regression models, after confirming that the assumptions of normality, linearity, and homoscedasticity were met through the inspection of histograms, P-P plots, and scatterplots. To describe facial shape, nine PCs of shape were used, explaining 70.2% of shape variation within the entire sample. Shape PCs were handled as the dependent variables in the multivariate regression models, whereas facial attractiveness and age were treated as independent variables. All statistical analyses were performed with ViewBox 4.1 software and IBM SPSS statistics for Windows (Version 29.0; Armonk, NY: IBM Corp.). The level of statistical significance was set at *p* = 0.05.

## Results

### Externally perceived facial attractiveness

Interrater reliability among the six examiners for facial attractiveness ratings was good (ICCA,6 = 0.79, 95% CI [0.74–0.83], *P* < 0.001). The average facial attractiveness score was 52.4 ± 11.0 VAS units in females and 52.2 ± 10.5 VAS units in males, with no difference between sexes. Similar results were observed in the primary subsample, which included White participants (Table 1). Comparisons with previously published data on self-perceived facial attractiveness of the same individuals [22] revealed that overall, all assessments were significantly more positive compared to externally perceived assessments, whereas males rated themselves as significantly more attractive than females (Table 1).

**Table 1:** Comparisons (independent t-tests) of self-perceived and externally perceived facial attractiveness scores within the entire sample and within subgroups

Group	Subgroup	N	Self-perceived attractiveness (VAS score)^1^			Perceived by others attractiveness (VAS score)			Self vs. Perceived by others attractiveness
			Mean	SD	P-value	Mean	SD	P-value	P-value
Entire sample	Females	393	63.60	12.90	< 0.001	52.36	11.00	0.871	< 0.001
	Males	208	67.57	13.20		52.20	10.47		< 0.001
Whites	Females	219	64.80	11.81	< 0.001	55.11	11.42	0.358	< 0.001
	Males	149	69.35	11.83		54.02	10.74		< 0.001

^1^Republished from: Kanavakis et al. [22]

### Shape analysis

**Fig. 2:**
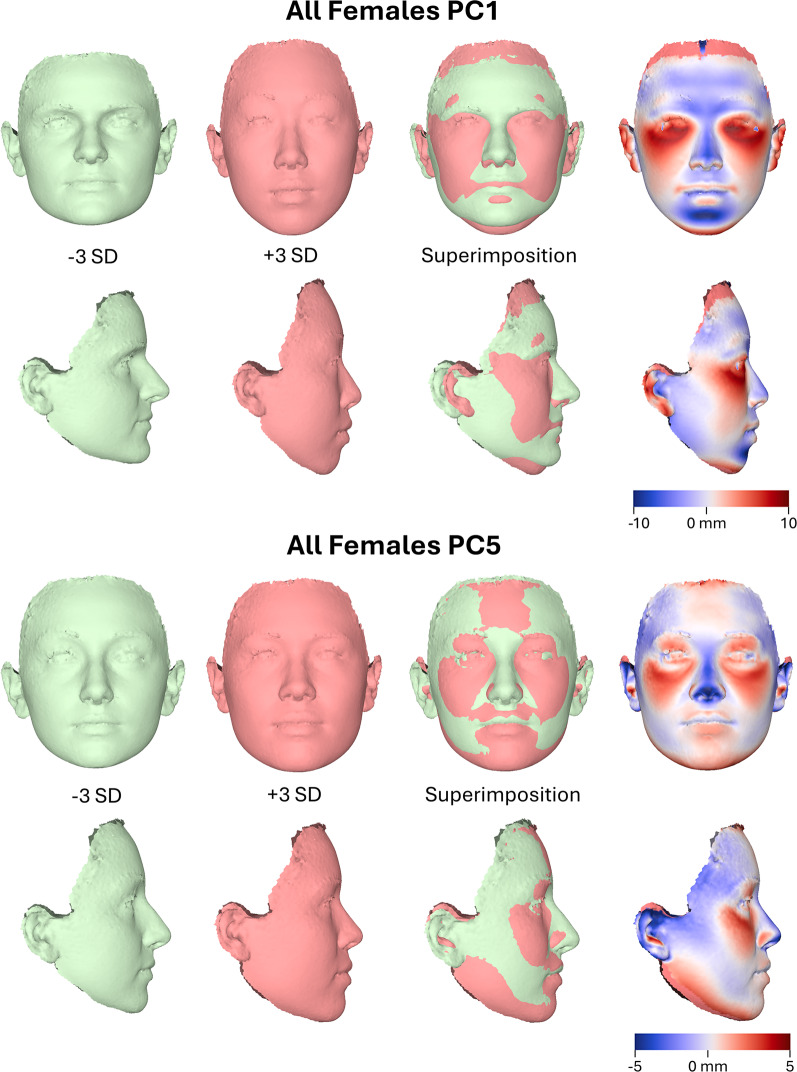
3D facial shape morphings of the average female shape configuration created from the Procrustes coordinates corresponding to -3SD (standard deviations) and +3SD of principal component (PC) scores along each axis. The variation explained by PC1 (21% of the total variation) and PC5 (4.9% of the total variation) is displayed. Best-fit superimpositions of corresponding frontal and profile shape transformations display the direction of shape variation explained by each PC (reference: -3SD)

A detailed assessment of overall facial shape variation in the sample has been presented previously [22]. In this study, which examines the association between facial shape and externally perceived attractiveness, we report the variation in principal components (PCs) that showed statistically significant associations with attractiveness. In the female sample, PC1 primarily reflects differences in facial width, profile convexity, chin projection, vertical facial dimension, eye shape, and midfacial projection. PC5 represents variation in the upper midface (infraorbital region), nasal base, and the vertical (downward) projection of the mandible (Fig. 2).

**Fig. 3:**
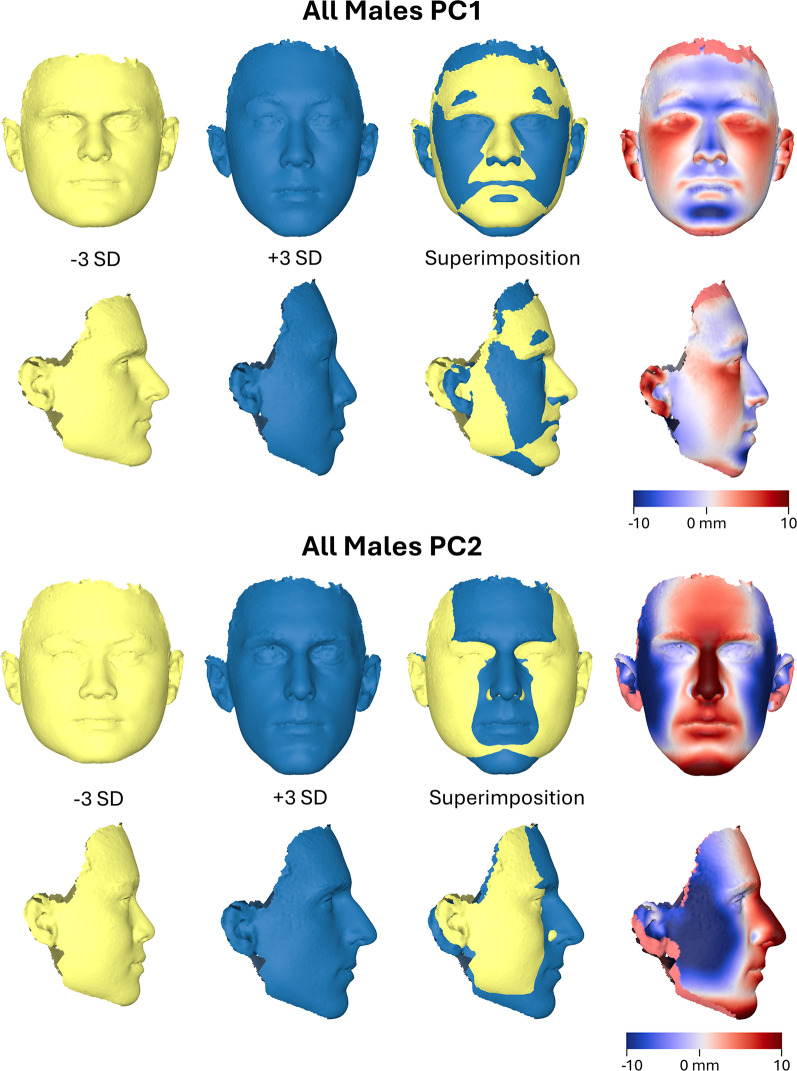
3D facial shape morphings of the average male shape configuration created from the Procrustes coordinates corresponding to -3SD (standard deviations) and +3SD of principal component (PC) scores along each axis. The variation explained by PC1 (20% of the total variation) and PC2 (16.8% of the total variation) is displayed. Best-fit superimpositions of corresponding frontal and profile shape transformations display the direction of shape variation explained by each PC (reference: -3SD)

Similarly, in the male sample, PC1 reflected variation in midfacial width, cheek and chin prominence, and vertical facial dimension. PC2 primarily captured differences in overall facial width, central facial projection—particularly of the nose—and chin prominence, along with vertical facial proportions. Both components also showed notable variation in eye and lip shape (Fig. 3).

### Facial shape and externally perceived facial attractiveness

1. Facial shape and facial attractiveness in females

**Fig. 4:**
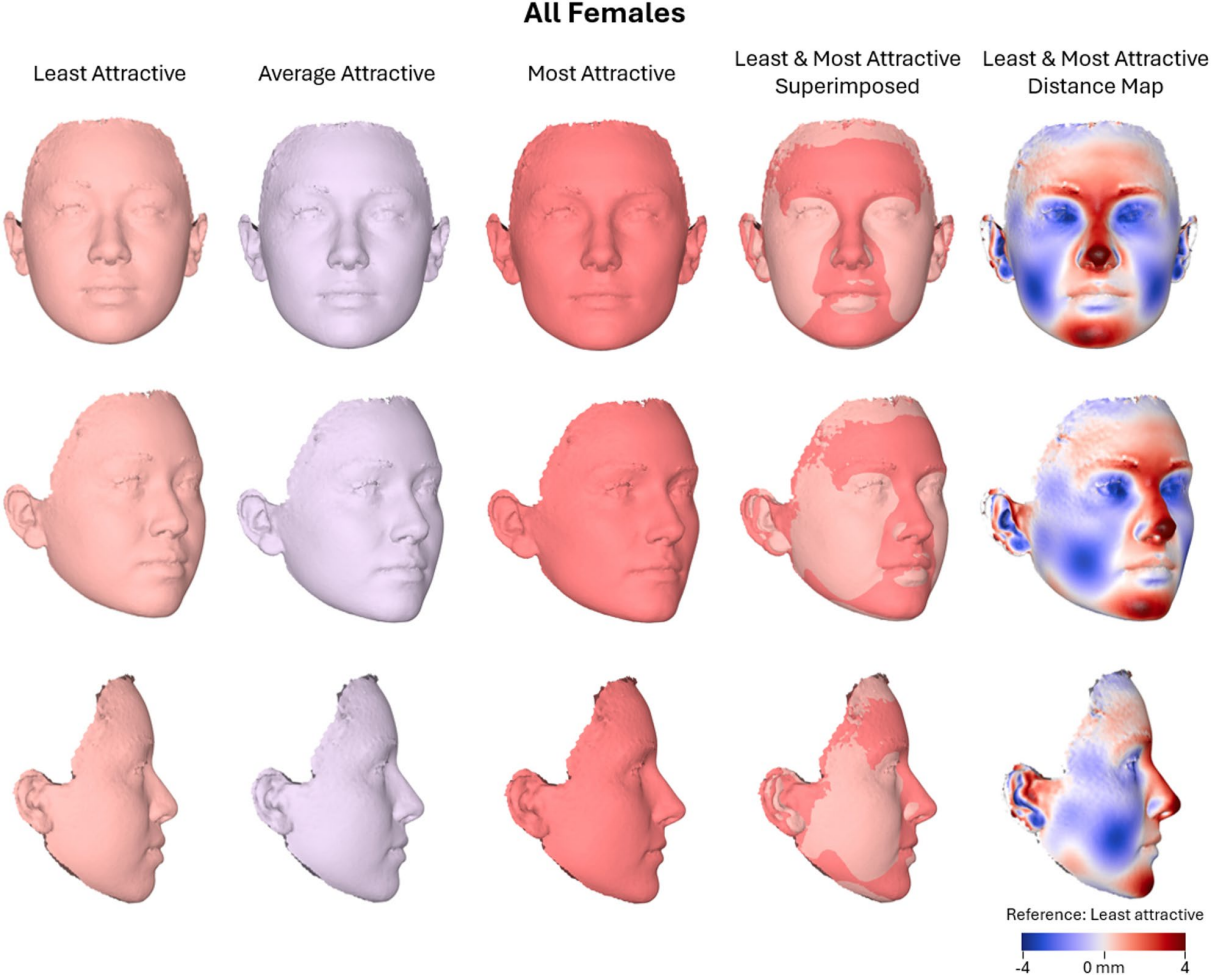
Warped facial images representing the least, average, and most attractive female faces (*n* = 393), along with a best-fit superimposition of the least and most attractive facial shapes. A color-coded distance map illustrates the surface differences between these two extremes, with color intensity indicating the magnitude and direction of displacement

Regarding the overall female sample (*N* = 393), the multivariate linear regression revealed a statistically significant association between facial shape and attractiveness ratings (Wilks’ Lambda = 0.925, F(9, 382) = 3.458, *P* < 0.001, partial η² = 0.075, observed power = 0.988), indicating a small to moderate effect size. Age was also significantly associated with facial shape (*P* = 0.003, partial η² = 0.063). Higher facial attractiveness was associated with slightly decreased facial width, reduced cheek volume, and more anteriorly projected central facial structures—most notably a narrower nose with a more protruded tip. Additionally, attractive faces featured a more prominent upper lip and a less retruded chin. Overall, increased attractiveness was linked to a more angular facial appearance, characterized by sharper contours and reduced facial fullness, with the exception of the upper lip, which appeared fuller and more projected (Fig. 4).

In the between-subjects tests, PC5 exhibited the strongest and statistically significant association with attractiveness ratings (F(1, 390) = 15.325, *P* < 0.001, partial η² = 0.038, observed power = 0.974), while PC1 also showed significant associations (F = 10.565, *P* = 0.001, partial η² = 0.026). All remaining components were non-significant, with small effect sizes (partial η² < 0.006) and low statistical power (observed power < 0.32). Adjusted R² values ranged from − 0.001 to 0.037 across all components, indicating that although significant, these effects accounted for a modest proportion of variation in facial shape. Overall, these results support a statistically reliable relationship between facial shape and perceived attractiveness in females, particularly along dimensions related to PC5 and PC1. PC5 primarily captured variation in the upper midface (infraorbital region), nose, and the vertical (downward) projection of the mandible, whereas PC1 captured variation related additionally to facial width, profile convexity, and chin projection (Fig. 2).

2. Facial shape and facial attractiveness in males

**Fig. 5:**
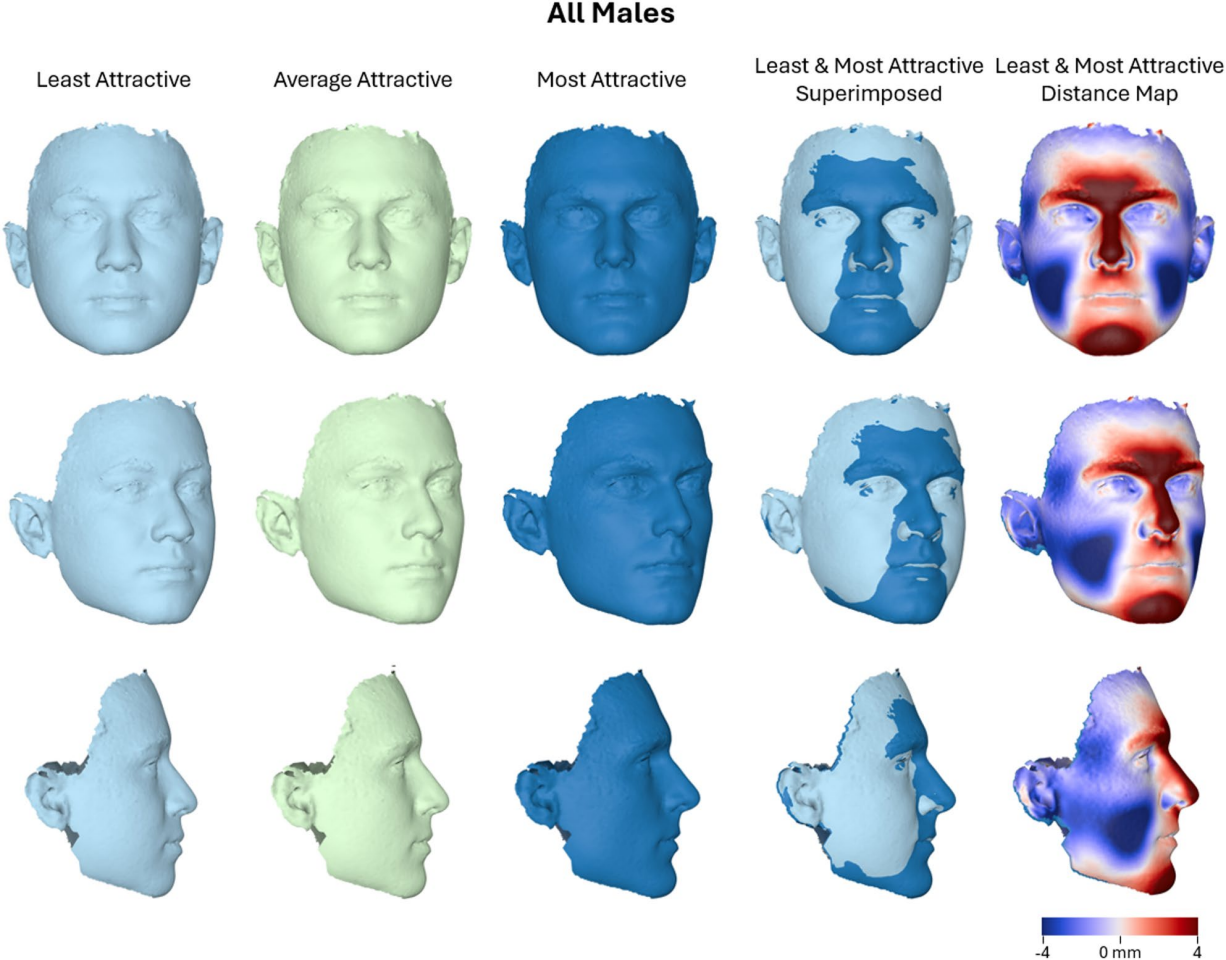
Warped facial images representing the least, average, and most attractive male faces (*n* = 208), along with a best-fit superimposition of the least and most attractive facial shapes. A color-coded distance map illustrates the surface differences between these two extremes, with color intensity indicating the magnitude and direction of displacement

Regarding the overall male sample (*N* = 208), the multivariate linear regression did not reach statistical significance for the association between facial shape and attractiveness ratings (Wilks’ Lambda = 0.930, F(9, 197) = 1.641, *P* = 0.106, partial η² = 0.070, observed power = 0.749), although the effect size was small to moderate and comparable to that observed in females. The effect of age was also not statistically significant (*P* = 0.532, partial η² = 0.039). Nonetheless, morphological differences between the least and most attractive male faces revealed certain consistent trends between sexes, as well as differences. As illustrated in Fig. 5, more attractive male faces tend to exhibit a narrower facial width, slightly longer face, reduced cheek volume, and more anteriorly projected central facial structures—particularly the nose, the chin, and the lower forehead, around the glabella. Additionally, the lip region and the lower mandibular contours appear more prominent, and the facial profile is straight. These features collectively contribute to a more angular and defined facial appearance, with sharper contours, decreased lateral fullness, and greater definition in the straight facial profile, particularly evident in the superimposed images and the distance map (Fig. 5). The shape differences associated with increased attractiveness were more pronounced in the male group than in the female group, as further illustrated in Supplementary Fig. 2.

In the between-subjects analysis, the strongest statistically significant associations with attractiveness ratings were found for PC1 (F(1, 205) = 6.626, *P* = 0.011, partial η² = 0.031, observed power = 0.726) and PC2 (F = 5.154, *P* = 0.024, partial η² = 0.025, observed power = 0.618). PC1 reflected variation in midfacial width, cheek and chin prominence, and vertical facial height, while PC2 primarily captured overall facial width, central facial projection—especially of the nose—and chin prominence, along with vertical proportions (Fig. 3). All other PCs had small effect sizes (partial η² < 0.005), low statistical power (observed power < 0.30), and were not statistically significant. Adjusted R² values across components ranged from − 0.009 to 0.025, suggesting that although specific components were statistically significant, they explained only a modest proportion of the variation in facial shape. Overall, the results suggest a marginally non-significant but visually and dimensionally consistent association between facial shape and perceived attractiveness in males, particularly in relation to PCs that reflect aspects of facial width, central face projection, and angularity.

3. White population (most represented in sample)

**Fig. 6:**
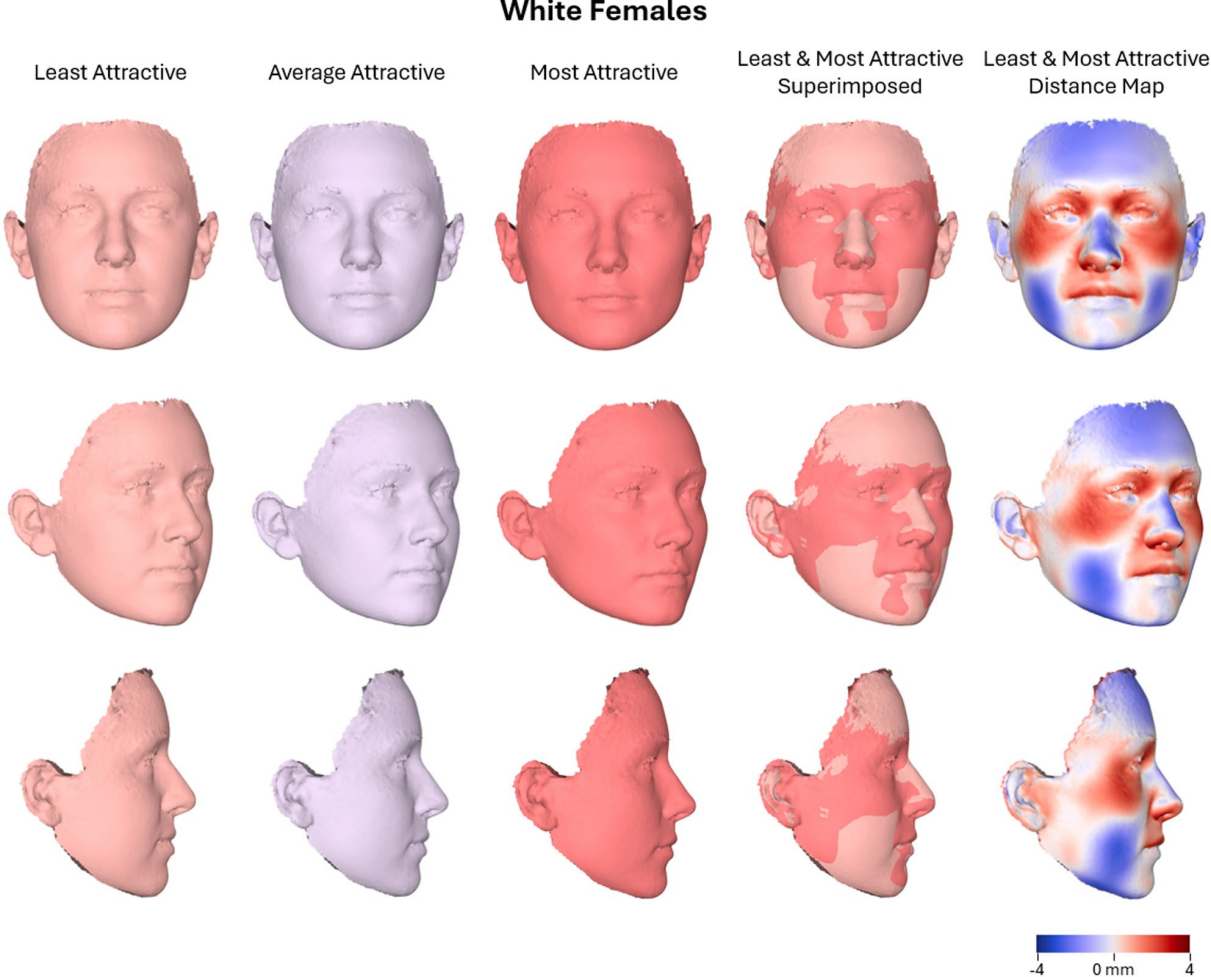
Warped facial images representing the least, average, and most attractive White female faces (*n* = 219), along with a best-fit superimposition of the least and most attractive facial shapes. A color-coded distance map illustrates the surface differences between these two extremes, with color intensity indicating the magnitude and direction of displacement

A multivariate linear regression was conducted to examine the association between facial shape and perceived facial attractiveness in the subsample of White females (*N* = 219), while controlling for age. The overall multivariate model approached statistical significance for attractiveness ratings (Wilks’ Lambda = 0.926, F(9, 208) = 1.85, *P* = 0.061, partial η² = 0.074, observed power = 0.811), indicating a small to moderate effect size. The effect of age was not significant (*P* = 0.132, partial η² = 0.063). In the between-subjects analysis, shape variation captured by PC4 showed a statistically significant association with attractiveness (F(1, 216) = 8.91, *P* = 0.003, partial η² = 0.040, observed power = 0.844). No other PCs showed significant associations. The adjusted R² for PC4 was 0.032, indicating that while the effect was statistically reliable, it explained a small portion of the variance in facial shape. These findings suggest that certain dimensions of facial shape are modestly related to perceived attractiveness in this group, even though the overall model narrowly missed conventional significance thresholds. More attractive White female faces are characterized by reduced buccal volume, a longer and thinner nasal dorsum and sidewall with a less deep Nasion area, increased anterior projection of the upper lip, and slightly shorter face. These changes result in a more angular and vertically proportioned facial appearance (Fig. 6).

**Fig. 7:**
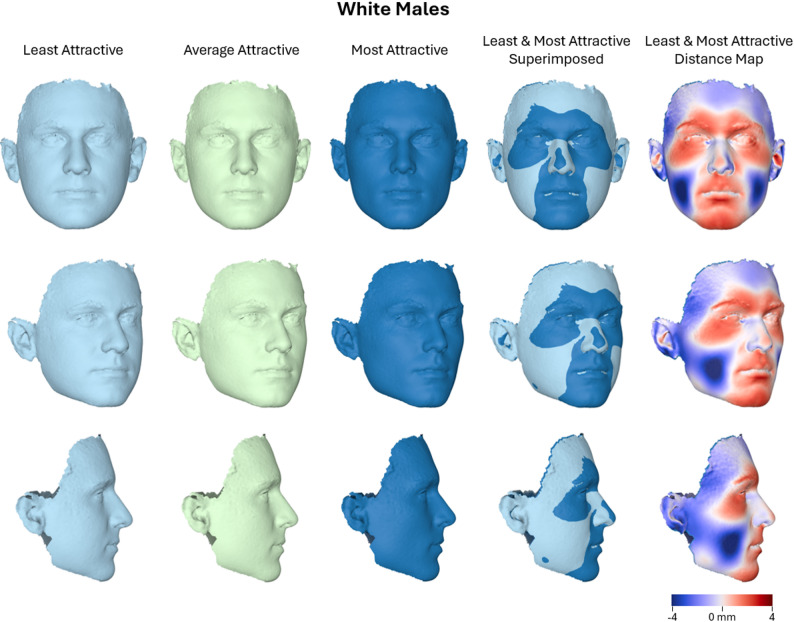
Warped facial images representing the least, average, and most attractive White male faces (*n* = 149), along with a best-fit superimposition of the least and most attractive facial shapes. A color-coded distance map illustrates the surface differences between these two extremes, with color intensity indicating the magnitude and direction of displacement

Regarding the White male subsample (*N* = 149), the overall multivariate model did not reveal a statistically significant association between facial shape and attractiveness ratings (Wilks’ Lambda = 0.970, F(9, 138) = 0.478, *P* = 0.888, partial η² = 0.030, observed power = 0.228). The effect of age was likewise non-significant (*P* = 0.361, partial η² = 0.067). In the between-subjects tests, none of the principal components demonstrated significant associations with attractiveness ratings. The component explaining the largest proportion of variance (PC6) showed a marginally higher effect size (partial η² = 0.031, F(2, 146) = 2.374, *P* = 0.097). All other components exhibited small effect sizes (partial η² < 0.012) and low power (observed power < 0.26). Adjusted R² values across all shape components ranged from − 0.013 to 0.018, indicating that the model explained very little variance in facial shape dimensions. Overall, these findings suggest no meaningful association between facial shape and attractiveness in this specific subsample, in contrast to the trends observed in the broader male sample. Although not statistically significant, the visual trends observed in this subsample were consistent with those observed in the full male sample (Fig. 7).

## Discussion

This study examined the association between three-dimensional facial shape and facial attractiveness as evaluated by external assessors and compared the findings with those from a prior investigation assessing self-perceived attractiveness on the same sample. Our findings suggest that objectively measurable aspects of facial shape influence attractiveness ratings, particularly in females. The integration of geometric morphometric methods with 3D surface data enabled a detailed and nuanced analysis of facial morphology and its relationship to perceived attractiveness.

In females, we observed a statistically significant association between facial shape and attractiveness ratings, with small to moderate effect sizes. More attractive female faces tended to have narrower facial width, reduced cheek volume, and greater projection of central facial structures, especially in the nasal and upper lip regions. These characteristics resulted in a more angular and less rounded appearance. These findings align well with those of Kanavakis et al. (2021) [22], where self-perceived facial attractiveness in females was also associated with a narrower face, fuller lower third anteriorly, and enhanced projection in the mid-forehead and nasal root. The results of that and the present study are directly comparable, since they consider the attractiveness of the same individuals. The consistency between external and self-perceptions in this group indicates a robust relationship between specific facial traits and attractiveness judgments, suggesting that females may be more sensitive to societal norms or more likely to internalize commonly accepted facial attractiveness standards [5, 45, 46].

The present findings align to some extent with existing evidence from 2D studies. For example, Przylipiak et al. (2018) [26] found a correlation between reduced nose proportions and increased attractiveness, particularly in females. Earlier studies have also suggested that typically feminine facial traits are perceived as more attractive in both female and male faces [17]. Here, a slightly narrower facial width, typically associated with femininity, was perceived as more attractive in both sexes. Findings regarding lip size are more controversial. While Przylipiak et al. (2018) [26] reported that smaller lips were perceived as more attractive, both the present study and the work of Horn et al. (2021) [23] found that greater lip projection was associated with higher attractiveness. This discrepancy may be attributed to the limitations of 2D imaging—especially when photographs are taken from varying angles—resulting in loss of depth and perspective. Additionally, the growing demand for lip fillers [47] and studies emphasizing femininity as a factor in perceived attractiveness [17] further support the notion that fuller lips are generally considered more attractive. A vertically increased lower third of the face has previously been associated with reduced attractiveness [27]. The current study demonstrates a positive correlation between a slightly longer lower third and facial attractiveness in males. These findings are not necessarily contradictory. It is possible that a vertically increased lower third is considered attractive up to a certain threshold, beyond which it may become less favorable. The aforementioned 2D studies used modified photographs of single models with few incremental changes to investigate features considered more attractive. In contrast, we used actual facial images of hundreds of participants and a more thorough approach to capture facial variation related to attractiveness.

In males, while the association between facial shape and externally perceived attractiveness showed comparable effect sizes to those in females (partial η² = 0.070 vs. 0.075), the lower sample size likely reduced statistical power, limiting the ability to reach conventional significance thresholds. The male sample was nearly half the size of the female sample (*N* = 208 vs. 393), with the corresponding p value indicating a trend toward significance (*p* = 0.106). This suggests that the lack of statistical significance may be more plausibly attributable to reduced power rather than absence of association. In addition, previous three-dimensional morphometric studies have reported greater inter-individual facial shape variability in males compared to females, which may further attenuate statistical detectability in smaller male samples [48]. Nonetheless, trends observed in morphological characteristics of more attractive male faces, such as reduced facial width, increased projection of the chin and central structures (particularly the nose and glabella), and overall increased angularity, mirrored those identified in the self-perception study. Specifically, Kanavakis et al. (2021) [22] reported that males rated themselves as more attractive when they had prominent chins, flatter cheeks, and enhanced projection of central facial features. Thus, although the statistical support was weaker in the externally perceived attractiveness model, the observed trends reinforce the notion that certain facial configurations are universally regarded—or perceived—as more favorable in males, both by the individual and society.

The difference in statistical significance between female and male samples may be partly attributed to variations in self-esteem and the influence of societal expectations. Prior research suggests that males often report higher self-esteem and are more lenient in self-assessments of attractiveness, potentially obscuring subtle shape-related associations in external assessments [49–51]. In our study, males consistently rated themselves as significantly more attractive than they were rated by others, whereas females displayed smaller discrepancies between self and external assessments. This might reflect a greater alignment between internal and external attractiveness criteria in females, or a higher self-critical standard due to sociocultural pressures.

Interestingly, in both self- and externally perceived assessments, certain morphological patterns recurred across sexes: increased midfacial projection, defined jawlines, and more angular profiles were common in more attractive faces. These features may represent culturally embedded standards of attractiveness, potentially influenced by evolutionary and hormonal cues linked to sexual dimorphism. While feminine traits such as lip fullness and narrower face were prominent in attractive females, the masculine correlates in males—such as prominent central facial structures, including chin projection—were also consistently observed.

When stratifying the analysis by ethnicity, results within the White female subgroup mirrored the overall findings, with specific principal components significantly associated with attractiveness ratings. These included traits such as reduced buccal volume and enhanced upper lip projection, consistent with the patterns observed in the self-perception study. The White female subgroup also represented the largest relatively homogeneous ethnic subgroup in the sample, which may have contributed to greater statistical stability. In contrast, neither the current nor the previous study identified significant associations in the White male subgroup. This absence of statistical significance may partly reflect the smaller male sample size, reducing statistical power. It is also conceivable that male attractiveness judgments may depend more on a broader constellation of cues beyond static facial shape, such as skin quality, coloration, and sexually dimorphic traits, which have been shown to contribute to perceived attractiveness [5, 52, 53]. This may reduce the proportion of variance explained by morphological components alone. Given the unequal distribution of participants across ethnic subgroups, these findings should be interpreted cautiously and considered exploratory.

Overall, our findings support the conclusion that facial shape significantly influences perceived attractiveness and that the traits identified align closely with those associated with higher self-perceived attractiveness. This concordance suggests that self-perceptions of facial attractiveness are not solely based on internal constructs but are also shaped by objective morphological traits that resonate with external societal standards. Clinically, this emphasizes the importance of facial morphology in perceived attractiveness and underscores the need for treatment planning approaches that account for both self-perception and social perception in aesthetic-focused interventions.

### Clinical implications

These findings carry meaningful implications for clinical disciplines that aim to enhance facial appearance, such as orthodontics [54, 55], plastic surgery, and maxillofacial surgery [56]. The consistent association between specific facial shape traits and perceived attractiveness underscores the importance of individualized morphological analysis in treatment planning. Contemporary digital tools, including three-dimensional soft-tissue prediction models and digital smile design protocols, allow clinicians to simulate modifications in central facial projection, transverse contours, and soft tissue balance. Integrating objective morphometric evidence into these digital workflows may enhance aesthetic outcome prediction and support patient-centered treatment planning. A holistic consideration of both self-perception and social perception of attractiveness should inform clinical decision-making and patient counselling.

### Strengths and limitations

A notable strength of this study is the use of a large and ethnically diverse sample with consistent sociocultural background, which enhances the generalizability of the findings within similar populations. The use of 3D surface imaging combined with geometric morphometrics provided a detailed and precise quantification of facial shape, enabling robust comparisons. Furthermore, the use of both self-perceived and externally assessed attractiveness data from the same individuals allowed for direct comparisons and deeper insight into perceptual dynamics.

However, certain limitations should be acknowledged. The study sample comprised highly educated young adults from a Western cultural setting, which may limit extrapolation to older or more socioeconomically diverse populations. Also, all evaluators were young adults with a background in health sciences. While this may have increased their sensitivity to aesthetic features, the intention of the panel was to estimate consensus perception under controlled conditions rather than to capture demographic variability in societal standards. The panel’s age composition is a strength of the study, as attractiveness perception is strongly age-dependent [57], and raters were therefore intentionally selected from the same age group as the participants to minimize age-related bias. Importantly, extensive facial-perception research has demonstrated substantial agreement in core attractiveness judgments across socioeconomic, cultural, and ethnic groups, particularly when evaluating structural facial characteristics such as symmetry, averageness, and sexual dimorphism [5, 58, 59]. The good interrater reliability observed in our study (ICC = 0.79), together with the alignment between external and self-perception findings, suggests that the panel captured a stable consensus signal rather than idiosyncratic demographic bias. A recent systematic review recommends approximately 10 evaluators for reliable assessment of facial aesthetics in individuals with cleft lip and palate [60]. Our study involved a large number of participants without specific morphological conditions. Although the number of evaluators was relatively small (*n* = 6), reliability was empirically assessed and found to be satisfactory. Previous methodological work on interrater reliability supports the adequacy of such designs when agreement indices are acceptable [61]. Nonetheless, broader and more demographically diverse rater panels would enhance generalizability in future studies. Finally, the present analysis does not account for dynamic facial features (e.g., expressions) or other important contributors to attractiveness such as skin texture, tone, and eye clarity.

Future research should aim to replicate these findings in more diverse populations and explore the contribution of dynamic and multimodal facial attributes to attractiveness perception.

## Conclusions

This study demonstrates that three-dimensional facial shape significantly influences externally perceived facial attractiveness, especially in females. Specific morphological traits, such as narrower facial width, reduced cheek volume, and increased projection of central facial structures like the nose and upper lip, were consistently associated with higher attractiveness ratings. These externally evaluated preferences closely mirror those seen in self-perceived attractiveness, suggesting a shared aesthetic standard and reinforcing the role of objective morphological features in the perception of beauty. While similar trends were observed in males, the associations did not consistently reach statistical significance, highlighting the possible influence of sociocultural or psychological factors on male attractiveness perceptions.

These findings underscore the importance of considering both objective facial traits and their perceptual impact in clinical contexts where facial aesthetics are addressed. Clinicians in orthodontics, maxillofacial surgery, and plastic surgery should incorporate such insights into treatment planning to align interventions with features that are positively perceived by both patients and their social environments.

## Supplementary Information

Below is the link to the electronic supplementary material.

Supplementary Material 1.

## Data Availability

All data are available in the main text or the extended data. The datasets generated and/or analysed during the current study will be available on request from the corresponding authors. Due to the sensitive nature of the used images, the raw data would remain confidential and would not be shared.

## Abbreviations

PC: Principal component
SD: Standard deviation
VAS: Visual analogue scale
ICC: Intraclass correlation coefficient
CI: Confidence interval (95% CI)
η²: Partial Eta-squared (effect size)
3D: Three-dimensional
2D: Two-dimensional
3dMD: 3D stereophotogrammetry system brand used for imaging
IRB: Institutional review board
SPSS: Statistical package for the social sciences
