# Supplementary material for: Influence of facial shape on perceived attractiveness
Source: Prog Orthod. 2026 Mar 25;27:13. doi: 10.1186/s40510-026-00617-2 (PMC13018507; doi:10.1186/s40510-026-00617-2)
Supplement: Supplementary file 1 — Supplementary Material 1. [file 40510_2026_617_MOESM1_ESM.pdf]

Supplementary material

Influence of Facial Shape on Perceived Attractiveness

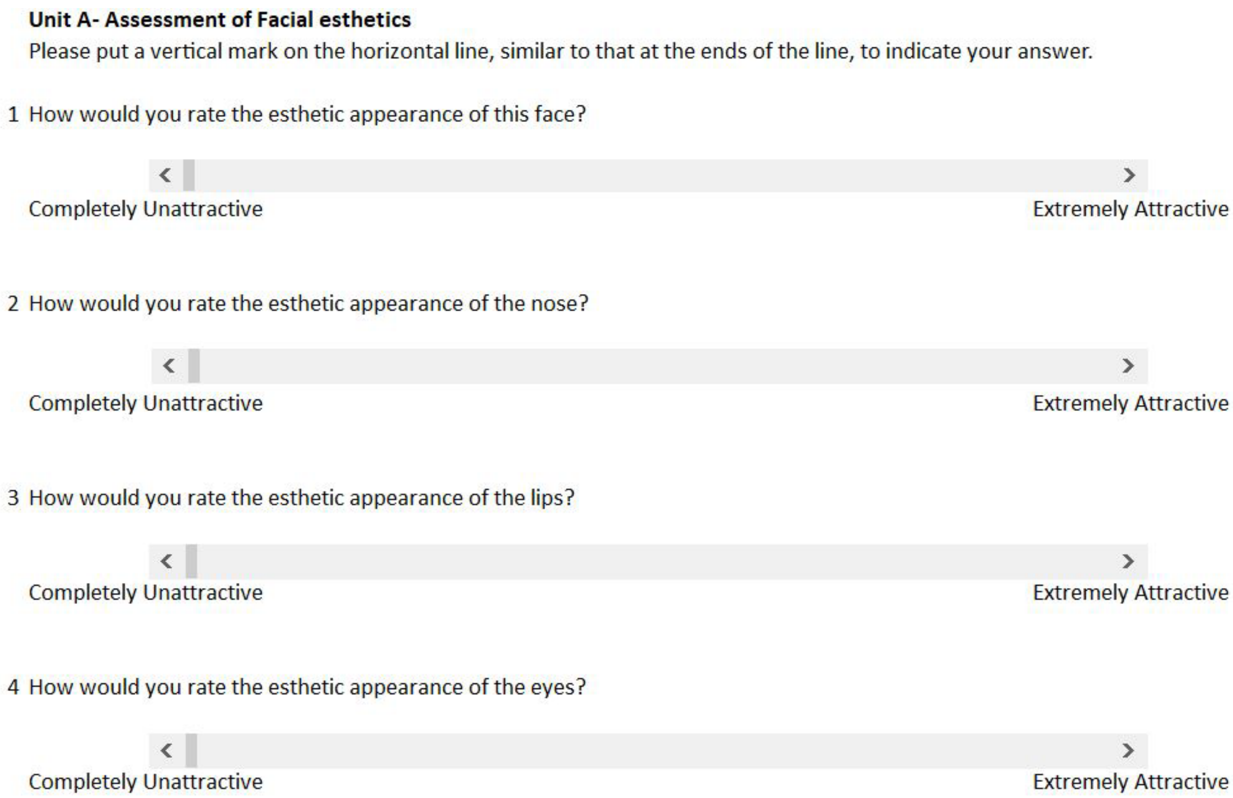

Unit A- Assessment of Facial esthetics

Please put a vertical mark on the horizontal line, similar to that at the ends of the line, to indicate your answer.

1 How would you rate the esthetic appearance of this face?

Completely Unattractive — Extremely Attractive

2 How would you rate the esthetic appearance of the nose?

Completely Unattractive — Extremely Attractive

3 How would you rate the esthetic appearance of the lips?

Completely Unattractive — Extremely Attractive

4 How would you rate the esthetic appearance of the eyes?

Completely Unattractive — Extremely Attractive

Supplementary Figure 1. Questionnaire used by the six evaluators for the assessment of facial attractiveness of the 601 participants.

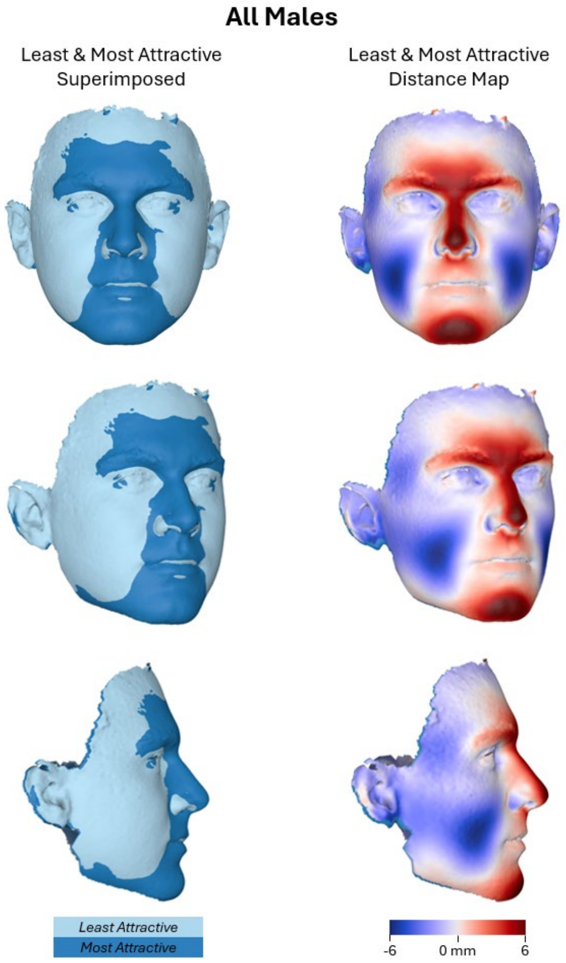

Supplementary Figure 2. Best-fit superimpositions of warped facial images representing the least and most attractive male faces (n = 208). A color-coded distance map illustrates the surface differences between these two extremes, with colour intensity reflecting both the magnitude and direction of displacement. This figure uses an increased displacement scale to enhance the visualization of surface changes; a version with a reduced scale is shown in the main text for consistency with the female data.
